# TRPC6-Mediated ERK1/2 Activation Increases Dentate Granule Cell Resistance to Status Epilepticus via Regulating Lon Protease-1 Expression and Mitochondrial Dynamics

**DOI:** 10.3390/cells8111376

**Published:** 2019-11-01

**Authors:** Ji-Eun Kim, Hana Park, Seo-Hyeon Choi, Min-Jeong Kong, Tae-Cheon Kang

**Affiliations:** 1 Department of Anatomy and Neurobiology, College of Medicine, Hallym University, Chuncheon 24252, Korea; jieunkim@hallym.ac.kr (J.-E.K.); M19050@hallym.ac.kr (H.P.); 20161239@hallym.ac.kr (S.-H.C.); kmj4180@hallym.ac.kr (M.-J.K.); 2 Institute of Epilepsy Research, College of Medicine, Hallym University, Chuncheon 24252, Korea

**Keywords:** dentate granule cell, epilepsy, hyperforin, LONP1, mitochondrial dynamics, neuroprotection, pilocarpine, seizure, siRNA

## Abstract

Transient receptor potential canonical channel-6 (TRPC6) is one of the Ca^2+^-permeable non-selective cation channels. TRPC6 is mainly expressed in dentate granule cell (DGC), which is one of the most resistant neuronal populations to various harmful stresses. Although TRPC6 knockdown evokes the massive DGC degeneration induced by status epilepticus (a prolonged seizure activity, SE), the molecular mechanisms underlying the role of TRPC6 in DGC viability in response to SE are still unclear. In the present study, hyperforin (a TRPC6 activator) facilitated mitochondrial fission in DGC concomitant with increases in Lon protease-1 (LONP1, a mitochondrial protease) expression and extracellular-signal-regulated kinase 1/2 (ERK1/2) phosphorylation under physiological conditions, which were abrogated by U0126 (an ERK1/2 inhibitor) co-treatment. TRPC6 knockdown showed the opposite effects on LONP1 expression, ERK1/2 activity, and mitochondrial dynamics. In addition, TRPC6 siRNA and U0126 evoked the massive DGC degeneration accompanied by mitochondrial elongation following SE, independent of seizure severity. However, LONP1 siRNA exacerbated SE-induced DGC death without affecting mitochondrial length. These findings indicate that TRPC6-ERK1/2 activation may increase DGC invulnerability to SE by regulating LONP1 expression as well as mitochondrial dynamics. Therefore, TRPC6-ERK1/2-LONP1 signaling pathway will be an interesting and important therapeutic target for neuroprotection from various neurological diseases.

## 1. Introduction

Mitochondria are essential organelles for cellular bioenergetics, which are responsible for producing nearly 95% of cellular ATP through oxidative phosphorylation. Under pathological conditions, a progressive decrease in the mitochondrial integrity abrogates respiratory capacities and increases production of free radicals, leading to aberrant structural and/or functional changes in mitochondria. Therefore, the maintenance of mitochondrial redox status is very important for cell viability [1,2,3,4,5].

Lon protease 1 (LONP1) belongs to the ATPases associated with diverse cellular activities (AAA+) protease family in the mitochondrial matrix that has a proteolytic activity of oxidized, dysfunctional, and misfolded proteins in ATP-dependent manner. Thus, LONP1 is rapidly up-regulated to prevent accumulation and aggregation of abnormal mitochondrial proteins under pathophysiological conditions [3,4,5,6,7]. LONP1 over-expression also activates extracellular signal regulated kinase 1/2 (ERK1/2), providing survival advantages and adaptation to cells [8]. Furthermore, ERK1/2 is required for the up-regulation of LONP1 during epidermal growth factor (EGF)-induced tumorigenic transformation [9]. Therefore, it is likely that the reciprocal regulation between ERK1/2 and LONP1 may affect neuron viability against harmful stresses, although the underlying mechanisms have been elusive. 

Transient receptor potential canonical channel-6 (TRPC6) is one of Ca^2+^-permeable non-selective cation channels, which protects neurons from ischemia [10], excitotoxicity [11], and status epilepticus (a prolonged seizure activity, SE) [12]. In the rat hippocampus, TRPC6 is highly expressed in the dentate granule cells (DGC), which are more resistant to various insults than other hippocampal neurons [13,14]. Furthermore, TRPC6 knockdown reduces ERK1/2 activity, and results in the massive DGC degeneration following SE [14,15,16]. Recently, we have reported that the abrogation of up-regulation of LONP1 expression by its siRNA evokes massive DGC death following SE [17]. Therefore, it is presumable that TRPC6-mediated ERK1/2 activation may be one of the up-stream signaling cascades that protect DGC from SE by regulating LONP1 expression, which is less defined.

Here, we show that TRPC6 knockdown led to mitochondrial elongation in DGC concomitant with decreases in LONP1 expression and ERK1/2 phosphorylation. Hyperforin (a TRPC6 activator) showed the reverse effects on ERK1/2 activity, LONP1 expression, and mitochondrial length. In addition, TRPC6 siRNA and U0126 (an ERK1/2 inhibitor) resulted in massive DGC degeneration following SE. However, LONP1 siRNA evoked SE-induced DGC degeneration without affecting TRPC6 expression, ERK1/2 phosphorylation, or mitochondrial morphologies. These findings for the first time demonstrate TRPC6-ERK1/2 activation may increase DGC invulnerability to SE by regulating LONP1 expression and mitochondrial dynamics.

## 2. Materials and Methods

### 2.1. Experimental Animals and Chemicals

Male Sprague–Dawley (SD) rats (7 weeks old) were used in the present study. Animals were kept under controlled environmental conditions (23–25 °C, 12 h light/dark cycle) with free access to water and standard laboratory food. All animal protocols were approved by the Administrative Panel on Laboratory Animal Care of Hallym University (Hallym 2018-2, April, 2018). All possible efforts were taken to avoid animals’ suffering and to minimize the number of animals used during the experiment. All reagents were obtained from Sigma-Aldrich (St. Louis, MO, USA), except as noted.

### 2.2. siRNA and Drug Infusion

Under Isoflurane anesthesia (3% induction, 1.5–2% for surgery, and 1.5% maintenance in a 65:35 mixture of N_2_O:O_2_), animals were stereotaxically implanted with a brain infusion kit 1 (Alzet, Cupertino, CA, USA) into the right lateral ventricle (1 mm posterior; 1.5 mm lateral; −3.5 mm depth to the bregma). The infusion kit was sealed with dental cement and connected to an osmotic pump (1007D, Alzet, Cupertino, CA, USA) containing (1) control siRNA, (2) rat TRPC6 siRNA, (3) rat LONP1 siRNA, (4) vehicle, (5) U0126 (a selective ERK1/2 inhibitor, 25 μM), (6) hyperforin (a TRPC6 activator, 6 μM), or (7) hyperforin + U0126 [12,15,16,18]. Rat TRPC6 siRNA and LONP1 siRNA sequences were 5’-GGAAUAUGCUUGACUUUGGAAUGUUUU-3’ [14] and 5’-GAGACAAGUUGCGCAUGAUTT-3’ [17], respectively. The non-targeting control siRNA sequence was 5’-GCAACUAACUUCGUUAGAAUCGUUAUU-3’. In a previous study and the present study, 50 µM of U0126 inhibited ERK1/2 phosphorylation in the hippocampus by ~50% after 7 days of over infusion [15]. An osmotic pump was placed in a subcutaneous pocket in the interscapular region. To measure the effect of each siRNA, U0126 or hyperforin on seizure susceptibility in response to pilocarpine, some animals were also implanted with a recording electrode (Plastics One, Roanoke, VA, USA) into the left dorsal hippocampus (−3.8 mm posterior; 2.0 mm lateral; −2.6 mm depth). Before an EEG recording, connecting wire and an electrode socket were inserted in an electrode pedestal (Plastics One, Roanoke, VA, USA). 

### 2.3. SE Induction and EEG Analysis

Three days after surgery, SE was induced by a single dose (30 mg/kg) of pilocarpine in rats pretreated (24 h before pilocarpine injection) with 127 mg/kg LiCl, as previously described [14,15]. Before pilocarpine injection, animals were given atropine methylbromide (5 mg/kg i.p.) to block the peripheral effect of pilocarpine. As controls, rats were treated with saline instead of pilocarpine. After injection, animals were monitored continuously for 2 h to register the extent of behavioral seizure activity. Behavioral seizure severity was also evaluated according to Racine’s scale [19]: (1) immobility, eye closure, twitching of vibrissae, sniffing, or facial clonus; (2) head nodding associated with more severe facial clonus; (3) clonus of one forelimb; (4) rearing, often accompanied by bilateral forelimb clonus; and (5) rearing with loss of balance and falling accompanied by generalized clonic seizures. Within 20–45 min of treatment with pilocarpine, animals became catatonic and began staring, followed by myoclonic twitching and often frequent rearing and falling. The behavioral seizure score reached 4–5 in all groups. There was no difference in the behavioral seizure score induced by pilocarpine among all the groups. In some animals, EEG signals were also recorded with a DAM 80 differential amplifier (0.1–3000 Hz bandpass, World Precision Instruments, Sarasota, TL, USA), digitized (sampling rates, 1000 Hz) and analyzed using LabChart Pro v7 (AD Instruments, Bella Vista, NSW, Australia). Total EEG power and spectrograms were automatically calculated in 2-hour recording session using a Hanning sliding window with 50% overlap [14,15]. Two hours after SE, animals received diazepam (Valium; Roche, France; 10 mg/kg, i.p.) to terminate SE.

### 2.4. Tissue Processing

Seven days after surgery (non-SE induced animals) or three days after SE, rats were perfused transcardially first with phosphate-buffered saline (PBS) followed by a fixative solution (4% paraformaldehyde in 0.1 M phosphate buffer, pH 7.4) during 30 min under urethane anesthesia (1.5 g/kg, i.p.). The brains were removed and submerged in the same fixative solution for 4 h at 4 °C. Following postfixation, brains were cryoprotected overnight in 30% sucrose solution (in 0.1 M PBS), and coronally sectioned with a cryostat at 30 μm, and consecutive sections were contained in six-well plates containing PBS. For western blot, animals were decapitated under urethane anesthesia (1.5 g/kg, i.p.). The hippocampus was rapidly removed and homogenized in lysis buffer. The protein concentration in the supernatant was determined using a Micro BCA Protein Assay Kit (Pierce Chemical, Rockford, IL, USA). 

### 2.5. Western Blot

Western blotting was performed according to standard procedures. Briefly, tissue lysate proteins were blotted onto nitrocellulose transfer membranes (Schleicher and Schuell BioScience Inc., Keene, NH, USA), then incubated with primary antibodies in Table 1. Immunoreactive bands were detected and quantified on ImageQuant LAS4000 system (GE Healthcare, Piscataway, NJ, USA). The values of each sample were normalized with the corresponding amount of β-actin as internal reference.

### 2.6. Immunohistochemistry and Fluoro-Jade B Staining

As previously described [14,15,16], free-floating sections were first incubated with 10% normal goat serum (Vector, Burlingame, CA, USA) in PBS for 30 min at room temperature. Sections were then incubated at room temperature for overnight in the mixture of primary antibodies (Table 1) in PBS containing 0.3% triton X-100 (Table 1). After three washes in PBS, sections were incubated for 1 h in fluorescein isothiocyanate (FITC)-, Cy3- or aminomethylcoumarin acetate (AMCA)-conjugated secondary antibodies (Vector, Burlingame, CA, USA). Sections (reacted with TRPC6 antibody only) were reacted with biotinylated secondary antiserum and avidin–biotin complex (Vector, Burlingame, CA, USA). Thereafter, immunoreactivity was developed by standard 3,3’-Diaminobenzidine reaction. The antibody that was preincubated with 1 μg of purified peptide (for TRPC6) or pre-immune serum was used as for negative control. To analyze the neuronal damage, we applied Fluoro-Jade B (FJB) staining (Histo-Chem Inc., Jefferson, AR, USA), according to the manufacturer’s instructions. Images were captured using an AxioImage M2 microscope or a confocal laser scanning microscope (LSM 710, Carl Zeiss Inc, Oberkocken, Germany) [14,15].

### 2.7. Cell Count and Measurement of Mitochondrial Length

As previously described [14,15], coronal images of the dentate gyrus (3–4 mm posterior to the bregma) were captured (15 sections per each animal) using 20× objectives, and areas of interest (1 × 10^5^ μm^2^) were selected from the dentate granule cell layer. Thereafter, FJB-positive neurons were counted on 20× images using AxioVision Rel. 4.8 Software. Individual mitochondrion in DGC (*n* = 20/section) was also captured using 63× or 100× objectives, and each length was measured by using AxioVision Rel. 4.8 Software or ZEN lite (Blue Edition, Carl Zeiss Inc., Oberkocken, Germany) software following 3D-reconstruction. Two different investigators who were blind to the classification of tissues performed cell counts and measurement of mitochondrial length.

### 2.8. Quantification of Data and Statistical Analysis

All data were analyzed using Student *t*-test, one-way ANOVA, or one-way repeated measure ANOVA to determine statistical significance. Bonferroni’s test was used for post hoc comparisons. A *p*-value below 0.05 was considered statistically significant.

## 3. Results

### 3.1. TRPC6 Knockdown and Hyperforin Reversely Regulate LONP1 Expression, ERK1/2 Phosphorylation, and Mitochondrial Length in DGC under Physiological Conditions

**Figure 1:**
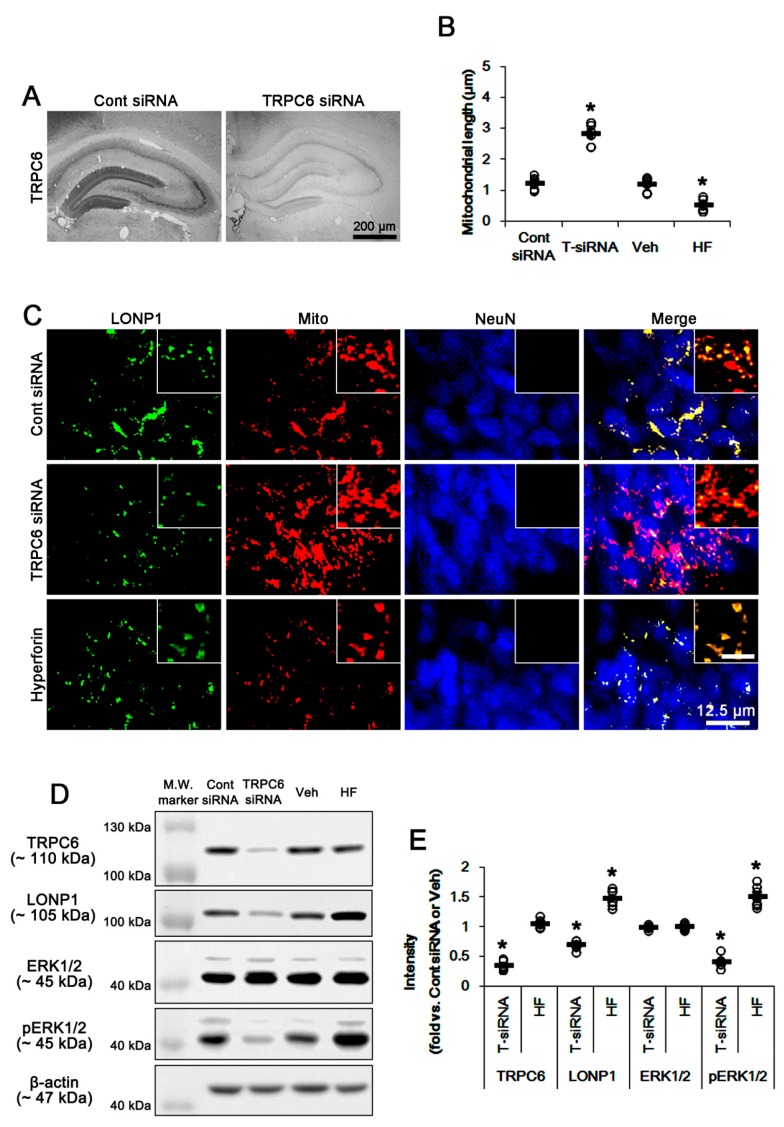
Effects of TRPC6 siRNA and hyperforin on mitochondrial dynamics, LONP1 expression, and ERK1/2 phosphorylation under certain physiological conditions. (**A**) Representative images of control- and TRPC6 siRNA-treated animals. TRPC6 expression is predominantly detected in the molecular layer of the dentate gyrus and DGC. TRPC6 siRNA effectively decreases TRPC6 expression. (**B**,**C**) Effects of TRPC6 siRNA and hyperforin on mitochondrial length. TRPC6 siRNA (T-siRNA) leads to mitochondrial elongation, while hyperforin (HF) facilitates mitochondrial fragmentation. (**B**) Quantification of mitochondrial length. Open circles indicate each individual value. Horizontal bars indicate mean value (mean ± S.E.M.; * *p* < 0.05 vs. control siRNA and vehicle, respectively; Student *t*-test; n = 7, respectively). (**C**) Representative double immunofluorescent images for LONP1 and mitochondria (Mito). Inserts are high magnification images (insert bar = 1.25 μm). (**D**,**E**) Effects of TRPC6 siRNA and hyperforin on expressions of TRPC6 and LONP1, and ERK1/2 phosphorylation. TRPC6 siRNA (T-siRNA) decreases protein levels of TRPC6 and LONP1, and ERK1/2 phosphorylation. Hyperforin (HF) increases LONP1 expression and ERK1/2 phosphorylation without changing TRPC6 expression. (**D**) Representative western blots of expressions of TRPC6 and LONP1, and ERK1/2 phosphorylation (M.W. marker, Molecular weight marker). (**E**) Quantification of expressions of TRPC6 and LONP1, and ERK1/2 phosphorylation based on western blot data. Open circles indicate each individual value. Horizontal bars indicate mean value (mean ± S.E.M.; * *p* < 0.05 vs. control siRNA and vehicle, respectively; Student *t*-test; n = 7, respectively).

Figure 1A shows that TRPC6 expression was apparently detected in the DGC layer and the molecular layer of the DG rather than other regions. TRPC6 siRNA reduced TRPC6 expression in the hippocampus (Figure 1A). TRPC6 knockdown elongated mitochondrial length (~2.86 μm), as compared to control siRNA (~1.26 μm) (*p* < 0.05 vs. control siRNA, n = 7; Figure 1B,C and Supplementary Figure S1). TRPC6 siRNA decreased LONP1 expressions in mitochondria (Figure 1C). Western blot data demonstrated that TRPC6 knockdown led to ~65% and ~30% reductions of TRPC6 and LONP1 protein levels, respectively (*p* < 0.05 vs. control siRNA, n = 7, respectively; Figure 1D,E). TRPC6 knockdown also declined ERK1/2 phosphorylation (*p* < 0.05 vs. control siRNA, n = 7; Figure 1D E and Supplementary Figure S2). In contrast to TRPC knockdown, hyperforin (a TRPC6 activator) [12,18] decreased mitochondrial length to ~0.54 μm (*p* < 0.05 vs. vehicle, n = 7; Figure 1B,C). Hyperforin increased ERK1/2 phosphorylation and LONP1 expression without altering TRPC6 expression (*p* < 0.05 vs. vehicle, n = 7; Figure 1D,E and Supplementary Figure S2). Since TRPC6 regulates ERK1/2 activity [20], our findings indicate that ERK1/2 may be involved in a potential relationship between TRPC6 and LONP1.

### 3.2. LONP1 siRNA Does Not Influence TRPC6 Expression, ERK1/2 Phosphorylation and Mitochondrial Length under Physiological Condition

**Figure 2:**
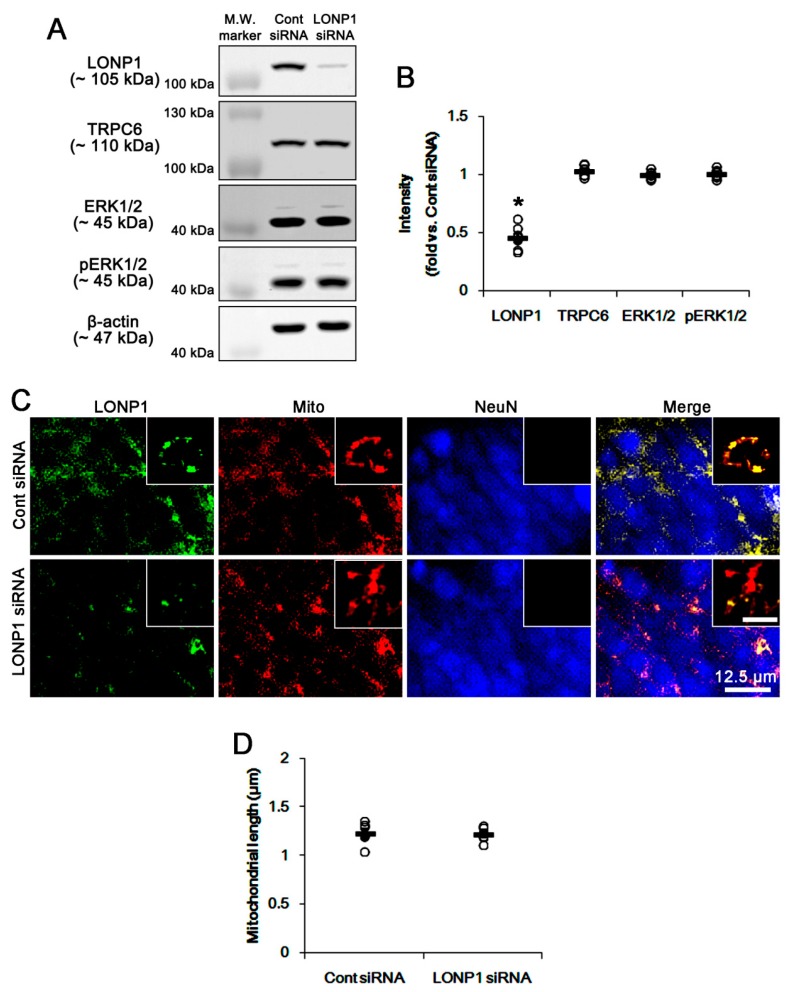
Effects of LONP1 siRNA on expression levels of LONP1 and TRPC6, ERK1/2 phosphorylation and mitochondrial dynamics under physiological condition. (**A**,**B**) Effects of LONP1 siRNA on expressions of TRPC6 and LONP1, and ERK1/2 phosphorylation. LONP1 siRNA decreases LONP1 expression without affecting TRPC6 expression and ERK1/2 phosphorylation. (**A**) Representative western blots of expressions of LONP1 and TRPC6, and ERK1/2 phosphorylation (M.W. marker, Molecular weight marker). (**B**) Quantification of expressions of LONP1 and TRPC6, and ERK1/2 phosphorylation based on western blot data. Open circles indicate each individual value. Horizontal bars indicate mean value (mean ± S.E.M.; * *p* < 0.05 vs. control siRNA; Student *t*-test; n = 7, respectively). (**C**,**D**) Effects of LONP1 siRNA on mitochondrial length. LONP1 siRNA does not affect mitochondrial length. (**C**) Representative double immunofluorescent images for LONP1 and mitochondria (Mito). Inserts are high magnification images (insert bar = 1.25 μm). (**D**) Quantification of mitochondrial length. Open circles indicate each individual value. Horizontal bars indicate mean value (mean ± S.E.M.; * *p* < 0.05 vs. control siRNA; Student *t*-test; n = 7, respectively).

Next, we applied LONP1 siRNA to confirm whether LONP1 reciprocally influences TRPC6 expression and ERK1/2 phosphorylation. LONP1 knockdown significantly decreased LONP1 expression (*p* < 0.05 vs. control siRNA, n = 7; Figure 2A,B and Supplementary Figure S3). However, LONP1 knockdown did not affect the TRPC6 expression level and mitochondrial length (Figure 2A–D and Supplementary Figure S1). In addition, LONP1 siRNA did not influence ERK1/2 expression and its phosphorylation (Figure 2A,B and Supplementary Figure S3). Thus, these findings suggest that the RPC6-ERK1/2 signaling pathway may be one of the up-steam regulators for LONP1 expression.

### 3.3. U0126 Abrogates Mitochondrial LONP1 Expression under Physiological Condition and after Hyperforin Treatment

**Figure 3:**
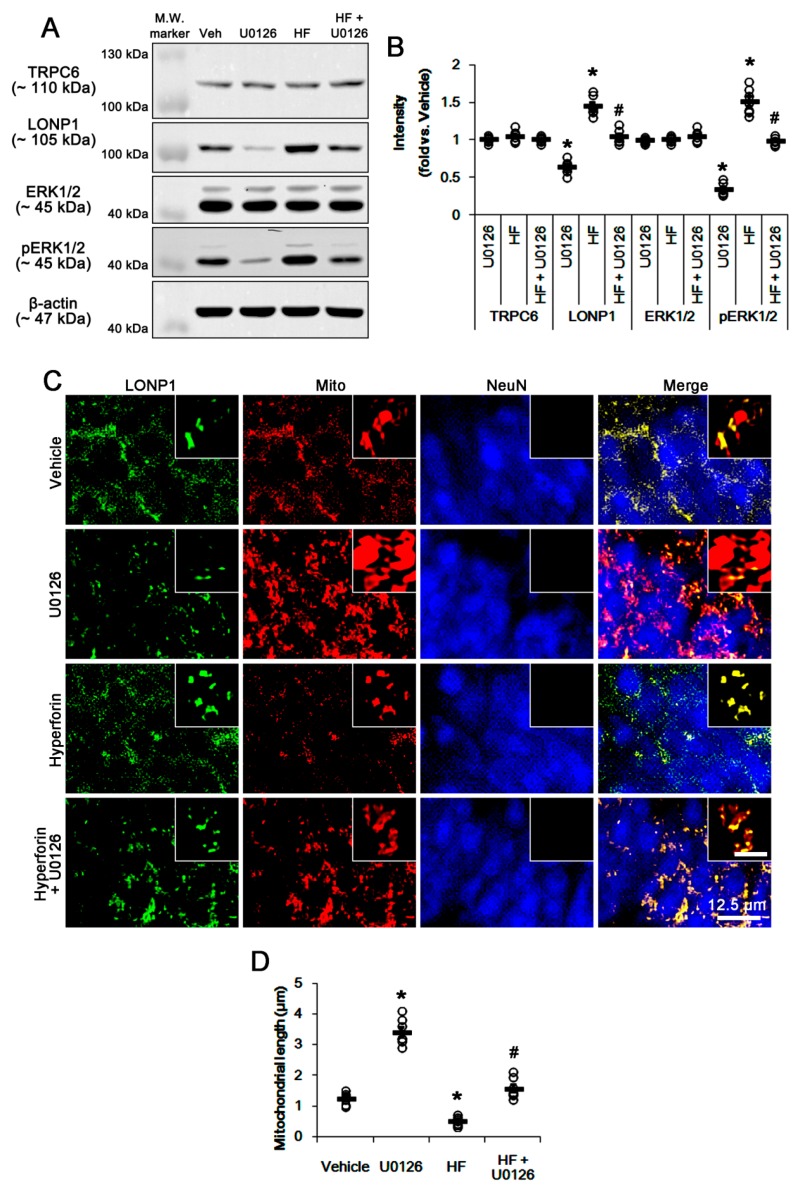
Effects of U0126, hyperforin, and co-treatment of hyperforin and U0126 on expression levels of LONP1 and TRPC6, ERK1/2 phosphorylation and mitochondrial dynamics under physiological condition. (**A**,**B**) Effects of U0126, hyperforin (HF) and co-treatment of hyperforin and U0126 (HF + U0126) on expressions of TRPC6 and LONP1, and ERK1/2 phosphorylation. U0126 decreases LONP1 expression and ERK1/2 phosphorylation without affecting TRPC6 expression. Hyperforin increases LONP1 expression and ERK1/2 phosphorylation, which are abrogated by U0126 co-treatment. (**A**) Representative western blots of expressions of TRPC6 and LONP1, and ERK1/2 phosphorylation (M.W. marker, Molecular weight marker). (**B**) Quantification of expressions of TRPC6 and LONP1, and ERK1/2 phosphorylation based on western blot data. Open circles indicate each individual value. Horizontal bars indicate mean value (mean ± S.E.M.; * *p* < 0.05 vs. control siRNA; one-way ANOVA; n = 7, respectively). (**C**,**D**) Effects of U0126, hyperforin (HF) and co-treatment of hyperforin and U0126 (HF + U0126) on mitochondrial length. U0126 increases mitochondrial length. In contrast, hyperforin diminishes it, which is abrogated by U0126 co-treatment. (**C**) Representative double immunofluorescent images for LONP1 and mitochondria (Mito). Inserts are high magnification images (insert bar = 1.25 μm). (**D**) Quantification of mitochondrial length. Open circles indicate each individual value. Horizontal bars indicate mean value (mean ± S.E.M.; * *p* < 0.05 vs. control siRNA; one-way ANOVA; n = 7, respectively).

Since hyperforin increases ERK1/2 phosphorylation and LONP1 expression in a previous [16] and the present study, we further investigated whether ERK1/2 activity affects LONP1 expression. U0126 (an ERK1/2 inhibitor) reduced ERK1/2 phosphorylation and LONP1 expression, and led to mitochondrial elongation under physiological condition without affecting TRPC6 expression (*p* < 0.05 vs. vehicle, n = 7; Figure 3A–D and Supplementary Figures S1 and S4). In addition, U0126 co-treatment abolished mitochondrial elongation and up-regulations of LONP1 expression as well as ERK1/2 phosphorylation induced by hyperforin (*p* < 0.05 vs. hyperforin, n = 7; Figure 3A–D and Supplementary Figures S1 and S4). Together with the data obtained from TRPC6 knockdown, these findings indicate that TRPC6 activity may regulate LONP1 expression and mitochondrial dynamics through ERK1/2 activation.

### 3.4. The TRPC6-ERK1/2-LONP1 Signaling Pathway Inhibits SE-Induced DGC Degeneration, Independent of Seizure Severity 

**Figure 4:**
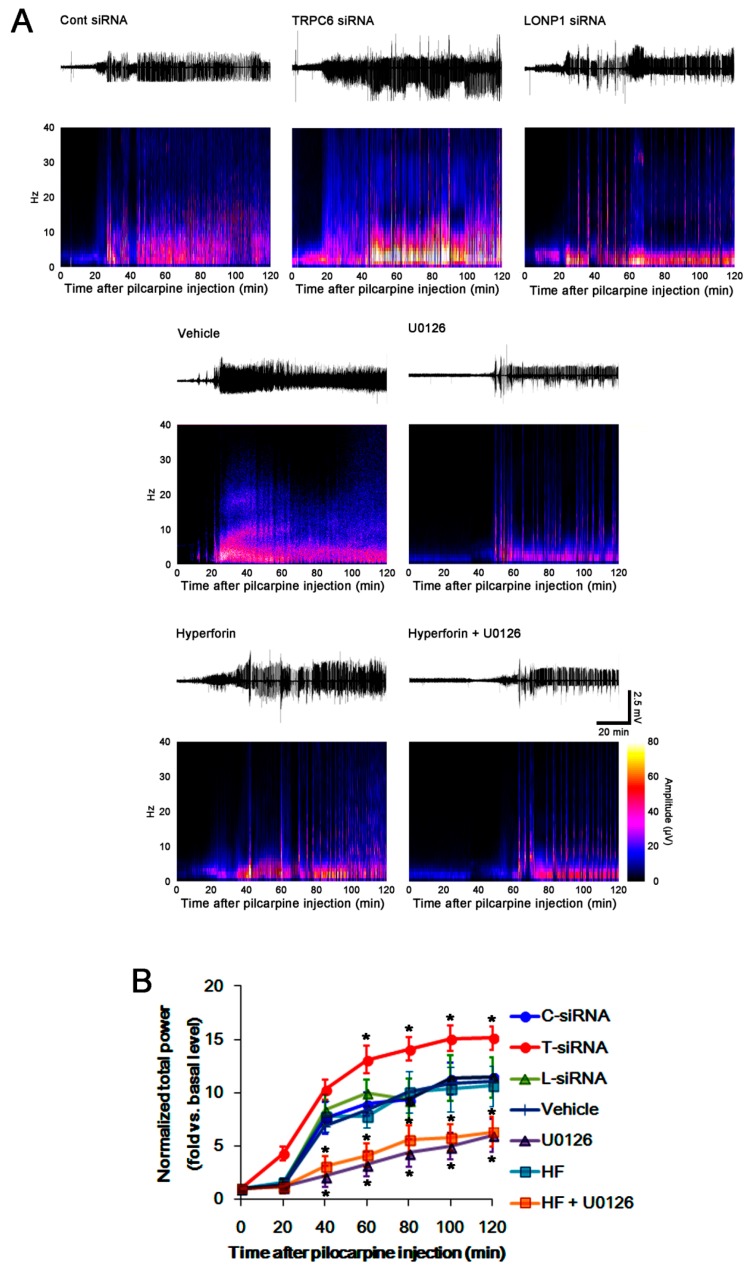
Effects of control siRNA, TRPC6 siRNA, LONP1 siRNA, U0126, hyperforin and co-treatment of hyperforin and U0126 on seizure activity in response to pilocarpine. As compared to control siRNA (C-siRNA), LONP1 siRNA (L-siRNA) does not affect seizure activity induced by pilocarpine. However, TRPC6 siRNA (T-siRNA) reduces seizure latency, and increases seizure severity in response to pilocarpine. No difference in seizure activity is observed between control siRNA and Vehicle (Veh)-treated animals. As compared to vehicle, hyperforin (HF) does not affect seizure activity induced by pilocarpine. However, U0126 and co-treatment of hyperforin and U0126 (HF + U0126) attenuate seizure activity in response to pilocarpine. (**A**) Representative EEG traces and frequency-power spectral temporal maps in response to pilocarpine. (**B**) Quantification of total EEG power (seizure intensity) in response to pilocarpine (mean ± S.E.M.; * *p* < 0.05 vs. control siRNA or vehicle; one-way repeated measure ANOVA; n = 7, respectively).

**Figure 5:**
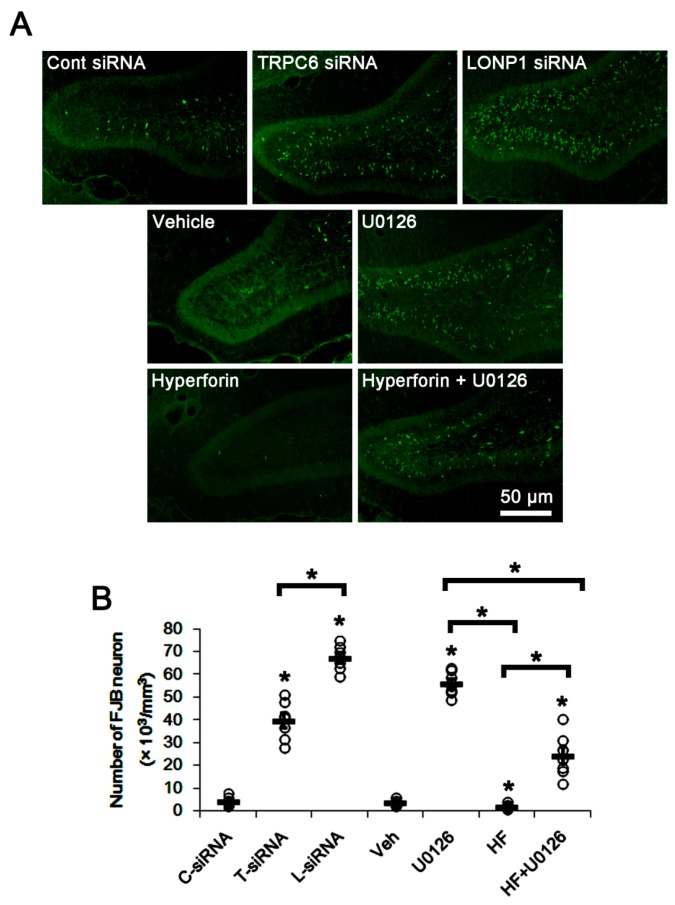
Effects of control siRNA, TRPC6 siRNA, LONP1 siRNA, U0126, hyperforin, and co-treatment of hyperforin and U0126 on SE-induced DGC degeneration. As compared to control siRNA (C-siRNA), TRPC6 siRNA (T-siRNA), LONP1 siRNA (L-siRNA) and U0126 induce massive DGC degeneration induced by SE. Hyperforin (HF) ameliorates SE-induced DGC damage, which is reversed by U0126 co-treatment (HF + U0126). (**A**) Representative images for FJB-positive degenerating DGC. (**B**) Quantification of the number of FJB-positive DGC. Open circles indicate each individual value. Horizontal bars indicate mean value (mean ± S.E.M.; * *p* < 0.05 vs. control siRNA or vehicle; one-way ANOVA; n = 7, respectively).

TRPC6 knockdown provokes massive DGC degeneration following pilocarpine-induced SE, although DGC is remarkably resistant to neuronal damage induced by various insults [14,15]. Since seizure severity correlates to neuronal damage [21,22], we explored whether the modulations of the TRPC6-ERK1/2-LONP1 signaling pathway alter seizure susceptibility to pilocarpine. In control siRNA-treated animals, the seizure susceptibility to pilocarpine was similar to that in vehicle-treated animals (Figure 4A,B). TRPC6 siRNA reduced the latency of seizure on-set, and increased total EEG power during SE (*p* < 0.05 vs. control siRNA, n = 7; Figure 4A,B). These findings indicate that TRPC6 knockdown may increase seizure susceptibility. LONP1 siRNA and hyperforin could not affect the seizure susceptibility to pilocarpine (Figure 4A,B). Consistent with our previous study [15], U0126 delayed the seizure on-set, and reduced total EEG power in response to pilocarpine (*p* < 0.05 vs. vehicle, n = 7; Figure 4A,B). Co-treatment of U0126 with hyperforin also reduced seizure activity after pilocarpine injection (*p* < 0.05 vs. vehicle, n = 7; Figure 4A,B). However, TRPC6 siRNA and LONP1 siRNA evoked massive DGC degeneration (*p* < 0.05 vs. control siRNA, n = 7; Figure 5A,B). As compared to vehicle, U0126 aggravated DGC death induced by SE (*p* < 0.05 vs. vehicle, n = 7; Figure 5A,B). Hyperforin attenuated SE-induced DGC degeneration (*p* < 0.05 vs. vehicle, n = 7; Figure 5A,B), which in turn caused deterioration by U0126 co-treatment (*p* < 0.05 vs. hyperforin, n = 7; Figure 5A,B). Therefore, the severity of SE-induced DGC degeneration in each siRNA or compound-treated animals was LONP1 siRNA > U0126 > TRPC6 siRNA > hyperforin + U0126 > control siRNA = vehicle > hyperforin. These findings suggest that the blockade of TRPC6-ERK1/2-LONP1 signaling pathway may increase SE-induced DGC degeneration, independent of seizure susceptibility or its severity.

## 4. Discussion

**Figure 6:**
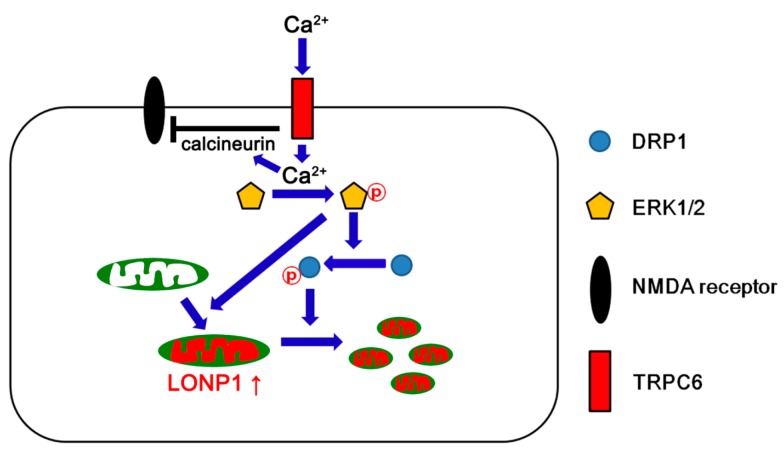
Scheme of roles of TRPC6 in LONP1 expression and mitochondrial dynamics based on the present data and previous reports [11,15,16,20,30]. TRPC6 activation increases Ca^2+^ influx in DGC. Intracellular Ca^2+^ activates calcineurin and ERK1/2. Activated calcineurin inhibits the NMDA receptor. In addition, ERK1/2 activation up-regulates LONP1 expression and DRP1 phosphorylation at the 616 site. Subsequently, phosphorylated DRP1 facilitates mitochondrial fission. Thus, TRPC6 may be involved in the quality controls of mitochondria as well as mitochondrial dynamics, which would enhance DGC invulnerability to SE.

The major findings of this study are that TRPC6-mediated ERK1/2 activation regulated LONP1 expression as well as mitochondrial dynamics, which were involved in the invulnerability of DGC to SE (Figure 6). 

LONP1 is an inducible ATP-stimulated protease, which plays important roles in cell viability by controlling the maintenance of mitochondrial homeostasis/bioenergetics and DNA integrity [7,23,24,25,26,27]. Therefore, LONP1 expression is up-regulated under some pathological conditions such as hypoxia, oxidative stress, and tumorigenesis [6,7,8,9]. However, the underlying mechanisms of regulation of LONP1 expression remain incompletely understood. TRPC6 modulates cell proliferation, differentiation and neuronal vulnerability to various insults [10,11,12,28,29]. In addition, TRPC6 activates ERK1/2 [15,30], which is involved in mitochondrial dynamics and LONP1 expression [9,31,32,33,34,35,36]. In the present study, we found that TRPC6 siRNA effectively reduced ERK1/2 activity (phosphorylation) and LONP1 expression under physiological conditions. In contrast, hyperforin, a TRPC6 activator [18,37], increased ERK1/2 activity and LONP1 expression, which were abrogated by U0126 co-treatment. Since LONP1 siRNA did not affect TRPC6 expression and ERK1/2 phosphorylation in the present study, our findings indicate that, at least in DGC, TRPC6-ERK1/2 signaling pathway is one of the up-stream regulators of LONP1 expression. 

LONP1 is required for the maintenance and expression of the mitochondrial enzymes and genomes [25,26,27]. In particular, LONP1 plays a direct role in the turnover of cytochrome c oxidase (COX), which is a terminal enzyme of the mitochondrial electron transport chain [38,39,40]. Under a hypoxic condition, LONP1 degrades isoform 1 of COX subunit 4 (COX4-1) to facilitate the switch from COX4-1 to COX4-2 for enhancing mitochondrial respiration [41]. LONP1 also removes the impaired human mitochondrial transcription factor A (TFAM) that is essential for mitochondrial DNA synthesis and its packaging [42,43,44]. Thus, deregulation of LONP1 leads to cell death by loss of mitochondrial functions [27,45,46]. In the present study, TRPC6 siRNA, LONP1 siRNA, and U0126 exacerbated SE-induced DGC degeneration. In addition, co-treatment U0126 abrogated the protective effect of hyperforin on DGC damage against SE. Therefore, our findings suggest that the TRPC6-ERK1/2 signaling pathway may play a neuroprotective role against SE by regulating LONP1-mediated mitochondrial homeostasis/bioenergetics. Further studies are needed to elucidate the specific targets controlled by LONP1, which would be involved in SE-induced neuronal death.

On the other hand, ERK1/2 activation accelerates mitochondrial fission via dynamin-related proteins 1 (DRP1)-serine (S) 616 phosphorylation [35,36]. Indeed, the blockade of TRPC6 functionality results in aberrant mitochondrial elongation by abrogating ERK1/2-mediated DRP1 activity in DGC [14,15]. Mitochondria are dynamic organelles responsible for generating ATP. In addition, mitochondrial dynamics participate in the synthesis of reactive oxygen species (ROS). Aberrant mitochondrial elongation inhibits mitochondrial respiratory function that triggers excessive ROS production. Excessive mitochondrial fission also impairs the detoxification of excess ROS and extrusion of intracellular Ca^2+^ [47,48]. Thus, imbalance of mitochondrial fission-fusion induces balance results in neuronal necrosis or apoptosis following SE [15,17,49,50,51,52,53]. Under physiological conditions, furthermore, mitochondrial fission directly enables increases mitochondrial ROS production [1,2]. Considering the relevance between mitochondrial dynamics and ROS syntheses, it is likely that the clearance of oxidized and misfolded proteins generated by ROS may be essential for cell viability. In the present study, TRPC6-mediated ERK1/2 activation facilitated mitochondrial fission, accompanied by LONP1 over-expression. However, LONP1 siRNA resulted in a massive DGC degeneration that was greater than the levels caused by TRPC6 siRNA and U0126, although it did not affect mitochondrial length. Unlike mitochondrial dynamics-related molecules (such as DRP1, optic atrophy 1, and mitofusin 2), LONP1 is up-regulated in response to harmful stresses [54]. Furthermore, LONP1 knockdown does not influence the activities of DRP1 and ERK1/2 under physiological- and post-SE conditions [17]. Since deregulation of LONP1 leads to cell death [17,27,54], the present data indicate that LONP1 may act as one of the important housekeeping antioxidants in mitochondria by limiting oxidative damage to tolerable levels, regardless of aberrant mitochondrial dynamics. Therefore, our findings suggest that TRPC6-ERK1/2-mediated LONP1 regulation may take part in the quality controls of mitochondria via degradation of oxidized/damaged proteins [23,24,25] and maintenance of mitochondrial DNA levels [27] during mitochondrial fission under physiological- and pathological conditions. 

In the present study, TRPC6 siRNA increased seizure susceptibility in response to pilocarpine. TRPC6 inhibits N-methyl-d-aspartate (NMDA) receptor activity mediated by calcineurin [11]. Indeed, TRPC6 knockdown increases the excitability ratio (an index of synaptic efficacy, also referred as excitatory postsynaptic potential-population spike amplitude coupling) [14,16] indicating the lowering intrinsic threshold of neuronal firing in postsynaptic neurons [55]. Therefore, TRPC6 knockdown reduces seizure threshold of DGC via the heightened efficacy of NMDA receptor function in DGC itself [16]. Furthermore, TRPC6 siRNA reduces ɤ-aminobutyric acid (GABA)-ergic inhibitions onto the DGC during and after high-frequency stimuli due to the impaired repetitive firing of interneurons [16]. However, the present data show that TRPC6 activation by hyperforin did not affect seizure susceptibility in response to pilocarpine. Unlike TRPC6 knockdown, hyperforin shows the distinct effects on evoked potentials in a dose-dependent manner. The higher concentrations of hyperforin (10 and 100 μM) reduce the population spike amplitude (an indicative of synchronous postsynaptic discharges [14,16]), while a lower concentration (1 μM) increases it [56]. Consistent with the present data, the concentration of hyperforin (6 μM) cannot affect GABAergic inhibition and the seizure susceptibility in response to pilocarpine due to the functional saturation of Kv4.3 channels in interneurons, unlike DGC [16,18]. Furthermore, Sell et al. [57] have reported that hyperforin induces TRPC6-independent H^+^ currents in HEK-293 cells, cortical microglia, chromaffin cells, and lipid bilayers. This action of hyperforin as a protonophore leads to cytosolic acidification and subsequently increases free intracellular Na^+^ concentration via Na^+^-H^+^ exchanger (NHE). Thus, it is plausible that this unspecific properties of hyperforin as protonophore may be also involved in the ineffectiveness of hyperforin on pilocarpine-induced seizure activity. This is because seizure activity results in biphasic pH shifts, consisting of an initial extracellular alkalinization, followed by a slower acidification. The early extracellular alkalosis increases excitability because of reductions in GABA_A_ receptor inhibition and enhancement in NMDA receptor currents, and the extracellular acidosis is involved in seizure termination [58]. Thus, it is presumable that hyperforin-induced H^+^ efflux from neurons or glia would attenuate seizure activity in response to pilocarpine, independent of TRPC6. However, the simultaneous Na^+^ accumulation would offset the inhibitory effect of extracellular acidosis on neuronal excitability by causing a lowering of the threshold for action potential generation in neurons and reducing the driving force for Na^+^-dependent re-uptake of glutamate and other excitatory neurotransmitters into glia or neurons [57,59,60,61]. Thus, it is likely that these discrepancies of hyperforin from TRPC6 siRNA may lead to the ineffectiveness of hyperforin on seizure susceptibility to pilocarpine in the present study.

## 5. Conclusions

The present data provide novel evidence that TRPC6 regulates LONP1 expression via ERK1/2 activity. In brief, TRPC6-mediated ERK1/2 activation increased LONP1 expression and facilitated mitochondrial fission. Thus, TRPC6 may be involved in the quality controls of mitochondria as well as mitochondrial dynamics, which would enhance DGC invulnerability to SE (Figure 6). To the best of our knowledge, the present study is the first indication of the role of the TRPC6-ERK1/2-LONP1 pathway in neuronal vulnerability to SE. Therefore, this signaling pathway will be an interesting and important therapeutic target for neuroprotection from various neurological diseases.

## Figures and Tables

**Table 1:** Primary antibodies used in the present study.

Antigen	Host	Manufacturer (Catalog Number)	Dilution Used
ERK1/2	Rabbit	Biorbyt (Orb160960)	1:2000 (WB)
LONP1	Rabbit	Proteintech (15440-1-AP)	1:100 (IF) 1:1000 (WB)
Mitochondrial marker (Mitochondrial complex IV subunit 1, MTCO1)	Mouse	Abcam (#ab14705)	1:500 (IF)
pERK1/2	Rabbit	Bioss (bs-3330R)	1:1000 (WB)
TRPC6	Rabbit	Millipore (AB5574)	1:100 (IHC) 1:1000 (WB)
β-actin	Mouse	Sigma (A5316)	1:5000 (WB)

IF, Immunofluorescence; IHC, immunohistochemistry; WB, Western blot.

## Supplementary Materials

The following are available online at https://www.mdpi.com/2073-4409/8/11/1376/s1, Figure S1: Representative photos of mitochondria for each siRNA or compound treated-animals. Figure S2: Full-length gel images of Western blot data in Figure 1D. Figure S3: Full-length gel images of Western blot data in Figure 2A. Figure S4: Full-length gel images of Western blot data in Figure 3A.
