# Supplementary material for: TRPC6-Mediated ERK1/2 Activation Increases Dentate Granule Cell Resistance to Status Epilepticus via Regulating Lon Protease-1 Expression and Mitochondrial Dynamics
Source: Cells. 2019 Nov 1;8(11):1376. doi: 10.3390/cells8111376 (PMC6912337; doi:10.3390/cells8111376)
Supplement: Supplementary file 1 [file cells-08-01376-s001.pdf]

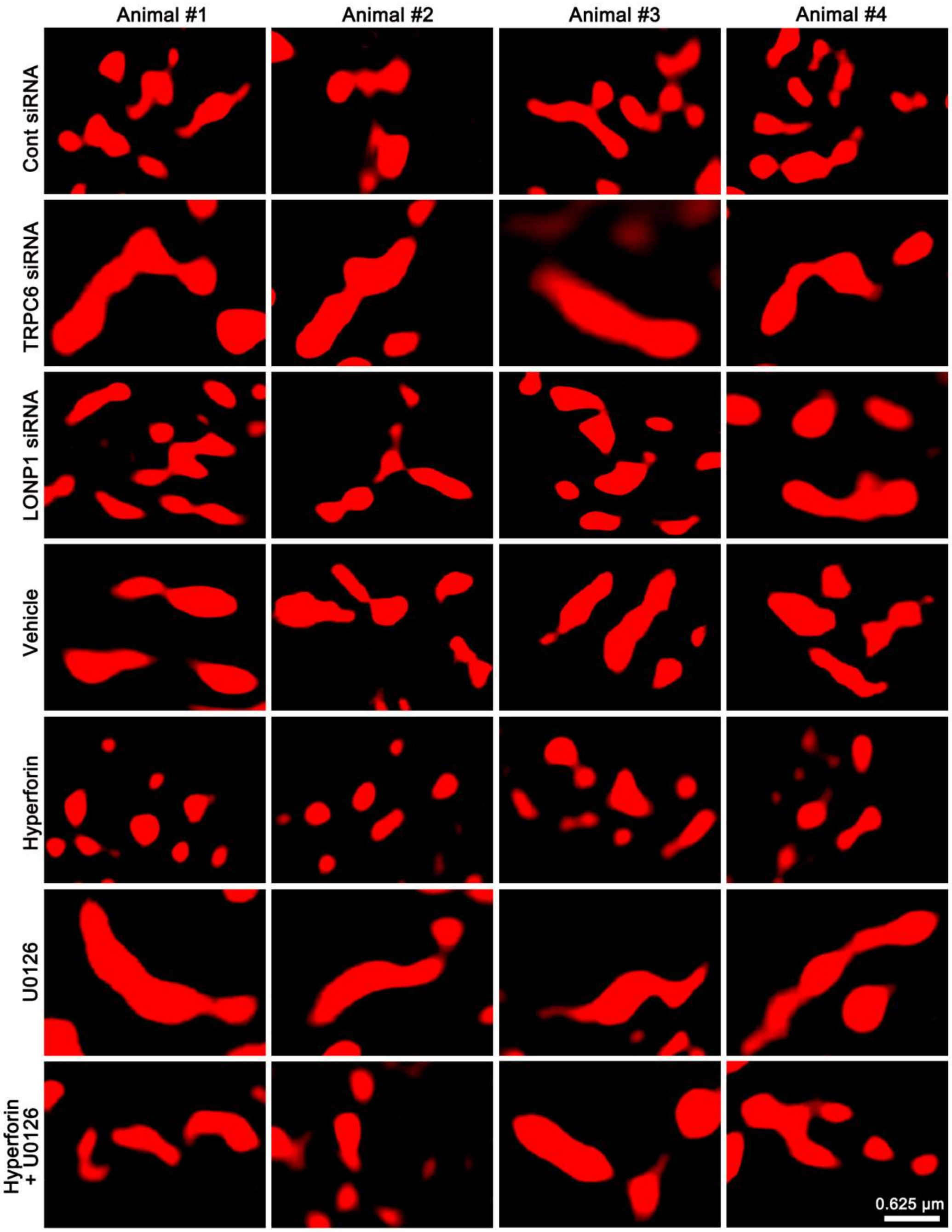

Supplementary Figure 1. Representative photos of mitochondria for each siRNA or compound treated-animals.

Fig. 1D

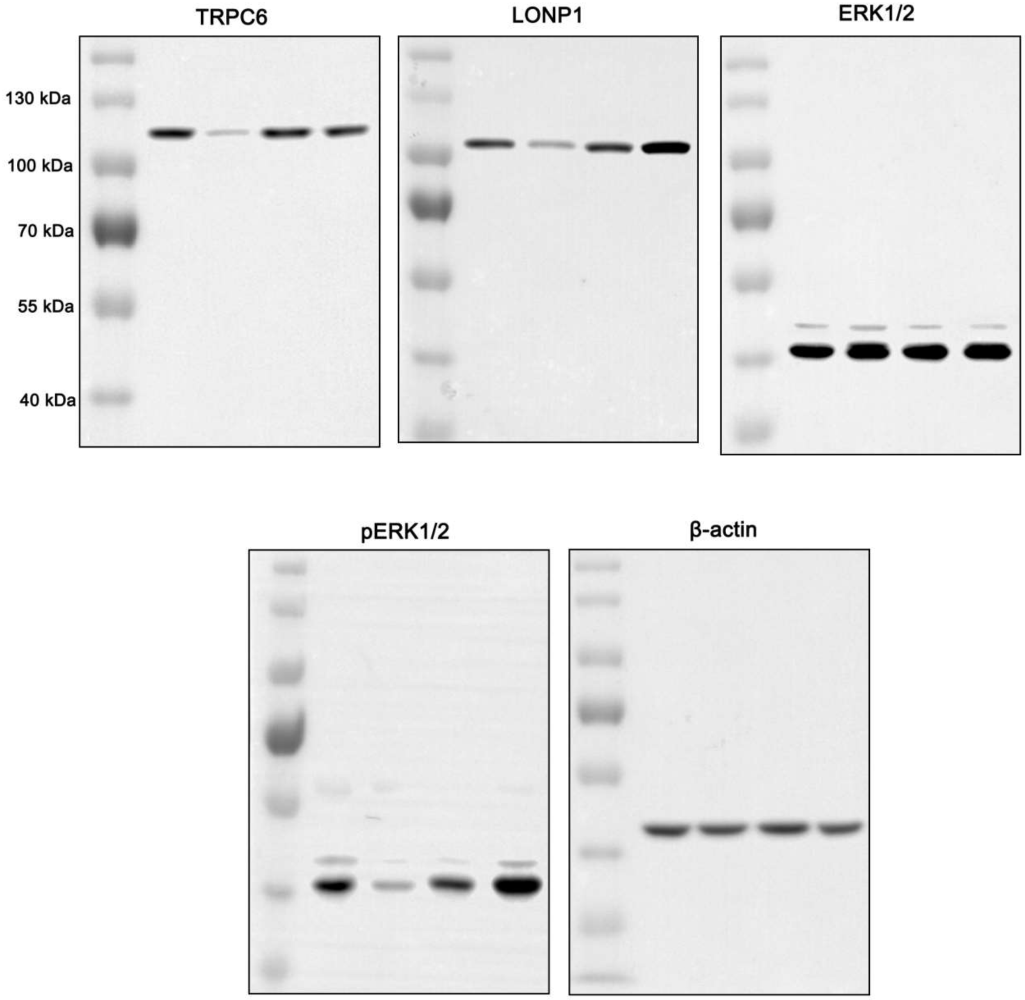

Supplementary Figure 2. Full-length gel images of Western blot data in Figure 1D.

Fig. 2A

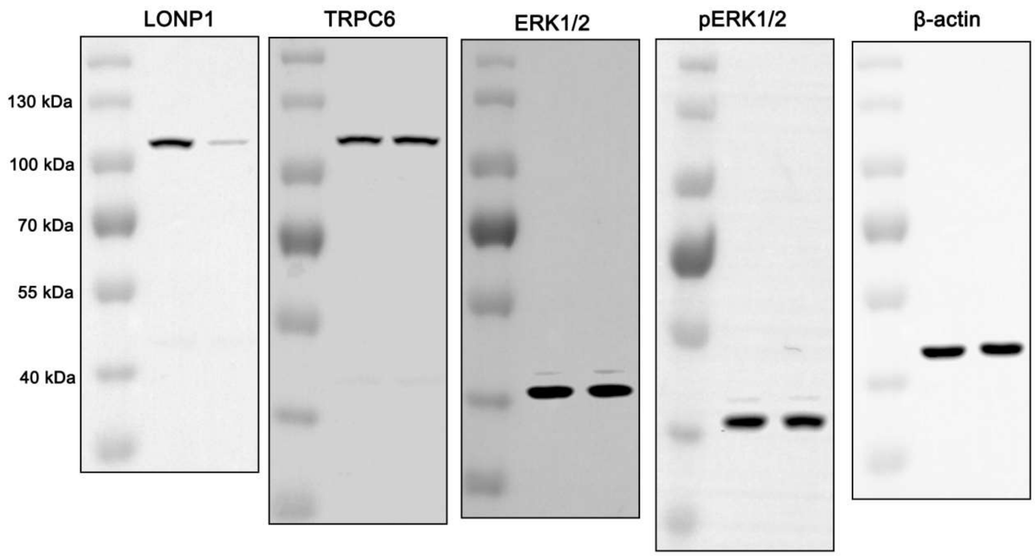

Supplementary Figure 3. Full-length gel images of Western blot data in Figure 2A.

Fig. 3A

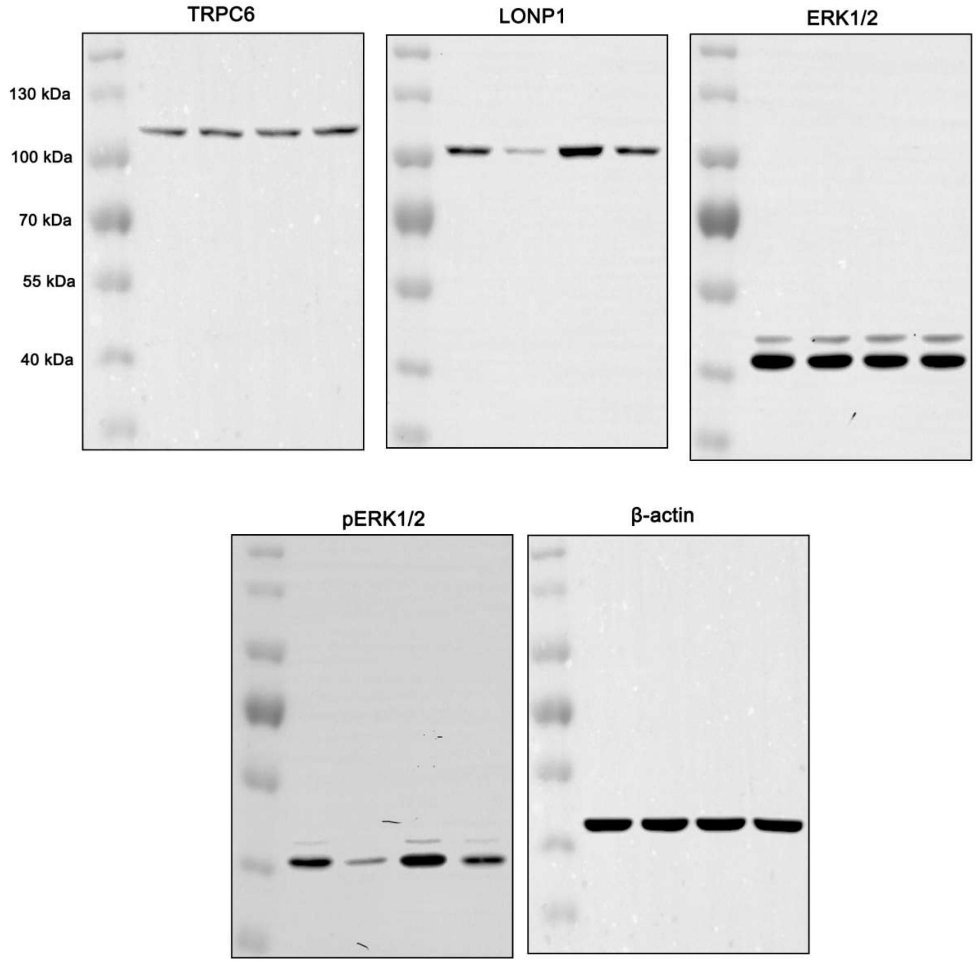

Supplementary Figure 4. Full-length gel images of Western blot data in Figure 3A.
